# Repurposing the Medicines for Malaria Venture’s COVID Box to discover potent inhibitors of *Toxoplasma gondii*, and *in vivo* efficacy evaluation of almitrine bismesylate (MMV1804175) in chronically infected mice

**DOI:** 10.1371/journal.pone.0288335

**Published:** 2023-07-07

**Authors:** Bruna Ramos dos Santos, Amanda Bruno da Silva Bellini Ramos, Renata Priscila Barros de Menezes, Marcus Tullius Scotti, Fábio Antônio Colombo, Marcos José Marques, Juliana Quero Reimão

**Affiliations:** 1 Laboratory of Preclinical Assays and Research of Alternative Sources of Innovative Therapy for Toxoplasmosis and Other Sicknesses (PARASITTOS), Departamento de Morfologia e Patologia Básica, Faculdade de Medicina de Jundiaí, Jundiaí, Brazil; 2 Departamento de Análises Clínicas e Toxicológicas, Faculdade de Ciências Farmacêuticas, Universidade Federal de Alfenas, Brazil; 3 Programa de Pós-graduação em Produtos Naturais e Sintéticos Bioativos (PgPNSB), Instituto de Pesquisa em Fármacos e Medicamentos (IPeFarM), Universidade Federal da Paraíba, João Pessoa, Brazil; University of Nairobi, KENYA

## Abstract

Toxoplasmosis, caused by the obligate intracellular parasite *Toxoplasma gondii*, affects about one-third of the world’s population and can cause severe congenital, neurological and ocular issues. Current treatment options are limited, and there are no human vaccines available to prevent transmission. Drug repurposing has been effective in identifying anti-*T*. *gondii* drugs. In this study, the screening of the COVID Box, a compilation of 160 compounds provided by the "Medicines for Malaria Venture" organization, was conducted to explore its potential for repurposing drugs to combat toxoplasmosis. The objective of the present work was to evaluate the compounds’ ability to inhibit *T*. *gondii* tachyzoite growth, assess their cytotoxicity against human cells, examine their absorption, distribution, metabolism, excretion, and toxicity (ADMET) properties, and investigate the potential of one candidate drug through an experimental chronic model of toxoplasmosis. Early screening identified 29 compounds that could inhibit *T*. *gondii* survival by over 80% while keeping human cell survival up to 50% at a concentration of 1 μM. The Half Effective Concentrations (EC_50_) of these compounds ranged from 0.04 to 0.92 μM, while the Half Cytotoxic Concentrations (CC_50_) ranged from 2.48 to over 50 μM. Almitrine was chosen for further evaluation due to its favorable characteristics, including anti-*T*. *gondii* activity at nanomolar concentrations, low cytotoxicity, and ADMET properties. Administering almitrine bismesylate (Vectarion®) orally at dose of 25 mg/kg/day for ten consecutive days resulted in a statistically significant (p < 0.001) reduction in parasite burden in the brains of mice chronically infected with *T*. *gondii* (ME49 strain). This was determined by quantifying the RNA of living parasites using real-time PCR. The presented results suggest that almitrine may be a promising drug candidate for additional experimental studies on toxoplasmosis and provide further evidence of the potential of the MMV collections as a valuable source of drugs to be repositioned for infectious diseases.

## Introduction

Toxoplasmosis is a disease that occurs in diverse ecosystems and has significant impacts on the health of humans, domestic animals, and wildlife. *Toxoplasma gondii*, the causative agent of toxoplasmosis, is an obligate intracellular parasite and one of the most well-adapted parasites in the world, capable of persisting for long periods in its hosts across different geographic regions [1].

Approximately one-third of the world’s population is affected by toxoplasmosis, a disease that can cause severe congenital neurological and ocular issues, especially in immunocompromised individuals [2]. The existing treatment regimen comprises only a limited number of medications, which can frequently lead to hypersensitivity and toxicity. Moreover, there is a notable absence of effective treatment options for chronic toxoplasmosis, highlighting the need for further research and development in this area [3].

Drug repurposing, also known as drug repositioning, involves identifying new uses for approved or investigational drugs that fall outside the scope of their original medical indication [4]. This approach has played a significant role in the discovery of anti-*T*. *gondii* drugs, offering several advantages such as potentially reduced development times and costs [5]. Sulfadiazine and pyrimethamine (PYR), both anti-*T*. *gondii* agents, are examples of repurposed drugs, originally developed to treat bacterial infections and malaria, respectively [6].

The “Medicines for Malaria Venture” (MMV) has successfully employed drug repurposing strategy by delivering its “MMV boxes” for their potential to be repurposed as effective treatments for different diseases beyond malaria. In the past few years, drug screenings of MMV collections against *T*. *gondii* have been conducted using the Malaria Box [7] and the Pathogen Box [8], both of which enabled the identification of potential novel drugs against the parasite.

In this study, we present, for the first time, the screening against *T*. *gondii* of the COVID Box, a MMV collection of 160 compounds with known or predicted activity against the coronavirus SARS-CoV-2. We evaluated the drugs’ ability to inhibit tachyzoite growth, their cytotoxicity against a human cell line, and the ADMET properties of the most selective compounds. Furthermore, we assessed the *in vivo* efficacy of almitrine bismesylate in treating a chronically infected experimental model of toxoplasmosis.

## Materials and methods

### Drugs and chemicals

Pyrimethamine (PYR), dimethyl sulfoxide (DMSO), chlorophenol red-β-D-galactopyranosidase (CPRG), phosphate buffer saline (PBS) and 3-[4,5-dimethylthiazol-2-yl]-2,5-diphenyltetrazolium bromide (MTT) were purchased from Sigma-Aldrich Corporation. Dulbecco’s Modified Eagle’s Medium (DMEM), fetal bovine serum (FBS), dithiothreitol (DTT), N-2-hydroxyethylpiperazine-N-2-ethane sulfonic acid (HEPES) and sodium dodecyl sulfate (SDS) were purchased from Thermo Fisher Scientific. The COVID Box was kindly donated by the Medicines for Malaria Venture (MMV) foundation. Almitrine bismesylate was purchased from CMS Científica do Brasil. Other analytical reagents were purchased from Sigma-Aldrich, unless otherwise stated.

### Cell culture and parasite propagation

To propagate *T*. *gondii* tachyzoites of the RH strain encoding a transgenic copy of β-galactosidase (type I, clone 2F1) [9], confluent monolayers of human foreskin fibroblasts (HFF) cells (ATCC, SCRC-1041) were continually passaged in DMEM supplemented with 2% FBS (D2 medium), L-glutamine (2 mM), and gentamicin (10 μg/mL) [10]. Freshly emerging tachyzoites were counted, diluted in fresh culture medium, and added to 96-well plates containing HFF cells (5 × 10^3^ parasites/well) as described below. All HFF and parasite cultures were incubated in a 37°C incubator supplemented with 5% CO_2_.

### Beta-galactosidase-based assay

The number of free tachyzoites of RH-2F1 *T*. *gondii* was estimated using a hemocytometer and a standard curve was prepared. Freshly harvested parasites were washed twice with phosphate-buffered saline (pH 7.2) and centrifuged at 1000g for 10 min. Different concentrations of tachyzoites ranging from 10^7^ to 2 parasites were used to obtain points for the standard curve. The β-galactosidase activity was determined as previously described [11]. Briefly, the parasites were incubated with 100 μL of lysis buffer (100 mM HEPES, 1 mM MgSO_4_, 0.1% Triton X-100, 5 mM DTT) for 15 min, followed by mixing the lysates with 160 μL of assay buffer (100 mM phosphate buffer pH 7.3, 102 mM β-mercaptoethanol, 9 mM MgCl_2_) and 40 μL of 6.25 mM CPRG. The reaction mixtures were incubated for 30 min, and the β-galactosidase activity was measured at 570 nm using a microplate reader (Thermo Scientific Varioskan LUX). Each parasite concentration was assessed in three replicate wells, and the presented data represent the results of two biological replicates.

### Anti-*T*. *gondii* growth inhibition assay

The 160 compounds of the COVID Box were supplied in 96-well plates. The 10 mM stock solution plates provided by MMV were diluted to a concentration of 1 mM using 100% DMSO. For the screening, the compounds were further diluted in the culture medium to achieve a final concentration of 1 μM in 96-well plates, following the instructions provided by the MMV Foundation. We determined the Half Effective Concentration (EC_50_) of compounds that inhibited parasite growth by at least 80% at a concentration of 1 μM, as previously described [12]. This approach allowed us to identify compounds with submicromolar activity and focus our analysis on these potent inhibitors. Firstly, 5 × 10^3^ HFF cells/well (in 100 μL volume) were placed in 96-well plates and incubated overnight to adhere. Then, the wells were emptied and refilled with fresh D2 medium containing 5×10^3^ RH-2F1 parasites (in 100 μL volume) and incubated for 3 h at 37°C with 5% CO_2_. Subsequently, the infected cells were incubated in the presence of drugs, assayed at a two-fold dilution, ranging from 2 μM to 15.62 nM, and incubated for 72 h at 37°C with 5% CO_2_. Each drug concentration was assessed in two replicate wells. Finally, the β-galactosidase activity was evaluated as described above. HFF-infected cells incubated in D2 without drug treatment were used as viability control, DMSO 2% was used as solvent control, wells without cells were used as negative control and pyrimethamine (1 μM) was used as a reference drug (positive control) in all assays. The data presented represent the results of two biological replicates. Dose-response inhibition curves (Log (inhibitor) vs. normalized response–variable slope) were obtained using Skanlt Software (Thermo Scientific, Waltham, MA, USA).

### Cytotoxicity in mammalian cells

The Half Cytotoxic Concentration (CC_50_) was assessed by using 96-well plates, for each compound inhibiting at least 80% of parasite growth at 1 μM. For this, HFF were seeded at 5 × 10^4^ cells/well in 96-well microplates and incubated over-night to adhere to the plate. Subsequently, cells were incubated in the presence of drugs (assayed at a two-fold dilution, ranging from 50 μM to 0.39 μM) for 72 h at 37°C in a 5% CO_2_ humidified incubator. The viability of the cells was determined by the MTT assay as previously described [13]. The medium in each well was replaced by PBS (100 μL/well), MTT (5 mg/mL) was added (20 μL/well), and the plate was incubated for 4 h at 37°C. Formazan extraction was performed using 10% SDS for 18 h (80 μL/well) at room temperature, and the optical density was measured at 550 nm using a microplate reader (Thermo Scientific Varioskan LUX). HFF incubated in D2 without drug treatment were used as viability control, DMSO 2% was used as solvent control, wells without cells were used as negative control and pyrimethamine (1 μM) was used as a reference drug (positive control) in all assays. Viability of 100% was expressed based on the optical density of untreated HFF cells, after normalization. The Selectivity Index (SI) was provided by the ratio between the CC_50_ against HFF cells and the EC_50_ against *T*. *gondii* tachyzoites. Data presented are representative of the results of two or more biological replicates. Dose-response inhibition curves (Log (inhibitor) vs. normalized response–variable slope) were obtained using Skanlt Software (Thermo Scientific).

### *In vivo* study

#### Animal maintenance and ethics statement

C57BL/6 mice were supplied by the animal breeding facility at the Universidade Federal de Alfenas. They were maintained in sterilized cages under a controlled environment and received water and food *ad libitum*. Animals chronically infected were euthanized in their home cage through CO_2_ inhalation one day after completion of drug treatment, on day 51 post-infection. The numbers of animals used was 16. No animal died before meeting criteria for euthanasia. Animal health and behaviour were monitored once a day. All animal welfare considerations were taken, including efforts to minimize distress. Besides, training in animal handling was provided for research equipe. Animal experiments were approved by the Ethics Committee for Animal Experimentation (Protocol 033/2021) of the Universidade Federal de Alfenas. The research adhered to the Brazilian Guidelines for Care and Utilization of Animals from the Conselho Nacional de Controle e Experimentação Animal (CONCEA).

#### Chronic infection

Chronic infection in Male C57/BL/6 mice (4 weeks-old) was obtained by intraperitoneal injection of brain tissue cysts (ME49 strain) as previously described [10]. The cysts were obtained by directly macerating the brain tissue of previously chronically infected mice in 5 mL of saline solution. The resulting mixture was then subjected to a counting procedure using a Neubauer chamber under a microscope to determine the concentration of the cysts. To prepare the cysts for injection, they were further diluted in saline solution to achieve the desired concentration of 10 cysts per animal. Subsequently, the mice in the experiment were infected with these freshly obtained brain cysts via intraperitoneal injection. The animals were considered chronically infected 40 days post-infection, according to the protocol described by [10]. Clinical parameters such as apathy, alopecia, and hair erection were observed to identify the chronic infection [14]. Additionally, the presence of brain cysts, known to be responsible for the observed symptoms, further confirmed the chronic infection status.

#### Evaluation of almitrine’s efficacy in infected mice

Forty days after infection, chronically infected mice were randomly assigned into experimental groups (n = 4 per group). These groups received 10 doses of almitrine bismesylate (25 or 12.5 mg/kg/day prepared with DMSO 4% in distilled water) or pyrimethamine (12.5 mg/kg/day prepared with distilled water) by oral gavage in 100 μL final volume. The control group received 100 μL of the vehicle used to dilute almitrine bismesylate (DMSO 4% in distilled water). On day 51 post-infection, all animals were euthanized, and their brains were collected for analyses. The brains were used for microscopic counting of cysts, measurement of cysts area, RNA extraction, and quantification. For microscopic counting, 25 μL of macerated organ was utilized, while 200 μL was used for RNA extraction. Cysts area was measured using ImageJ software, available at https://imagej.nih.gov/ij/.

#### Standard curve construction

We performed a serial dilution of cultured tachyzoites of *T*. *gondii* (RH strain), ranging from 5×10^6^ to 7.8×10^4^ forms per mL. DNA samples were extracted from each point of the serial dilution (in duplicate samples), and real-time PCR was performed. The resulting data were used to plot the cycle threshold value (C_T_) against varying amounts of parasites, ultimately generating a standard curve. The parasite burden of the *in vivo* experiment was subsequently estimated by analyzing the brain samples. The C_T_ values were utilized to determine the number of parasites in each sample through linear regression analysis. To accomplish this, we employed the equation **y = a + bx** (where **y** represents the C_T_ value, **a** denotes the y intercept, **b** signifies the slope, and **x** represents the number of parasites). By utilizing the linear regression data from tachyzoites, we were able to accurately calculate the number of parasites present in each animal sample [15].

#### DNA and RNA extractions and c-DNA synthesis

DNA and RNA samples were obtained from cultured tachyzoites and from brain fragments collected from treated mice. DNA extraction and purification were done using Pure-Link Genomic DNA Minikit (Invitrogen), according to the manufacturer’s instructions. RNA was extracted and purified from the brain of experimental animals using Reliaprep RNA Tissue Miniprep System (Promega), according to the manufacturer’s instructions. Immediately after extraction, RNA samples were processed for cDNA synthesis, following the steps: mix 1 was prepared with 11μL of extracted RNA, 1μL of DNTP and 1μL of random primers; mix 2 was prepared with 2μL of DTT and 4μL of buffer (Tris–HCl, 250 mM, pH 8,3; KCl, 375 mM; MgCl_2_ 15 mM). For reverse transcription, M-MLV RT (Moloney Murine Leukemia Virus Reverse Transcriptase) was used. In the end, 20μL of cDNA was synthetized and stored at -20°C for future qPCR analysis.

#### Primer selection and real time PCR

Each DNA and cDNA samples were analysed by quantitative real-time PCR using the set of primers Rep529, which amplified a *T*. *gondii* repetitive DNA fragment [16]. The sequences are as follows: forward primer: 5’-GTT GGG AAG CGA CGA GAG TC-3’; reverse primer: 5’-ATT CTC TCC GCC ATC ACC AC-3’; TaqMan probe FAM dye-labelled: 5’-AGA AGA TGT TTC CGG CTT GGC TGC TT-3’ and NFQ as quencher. Reactions were performed with Applied Biosystems™ StepOne™ Real-Time PCR System, using 3,5μL of PCR-water, 5μL of 2X Universal TaqMan Master Mix, 0,25μL of “primer mix” (0,5μL of both forward and reverse primers and 0,5μL of probe) and 1μL of sample. Amplification runs contained a negative control (reaction mix without DNA) and a positive control (high concentrated sample from standard curve). Thermal profiles were 2 min, 50°C, and 95°C for 10 min, then 40 cycles were performed at 95°C for 15 s and 60°C for 1 min. Reactions’ standardization was previously done with templates with different concentrations of *T*. *gondii* DNA. Data on parasite burden in the brain on infected mice was analysed for statistical significance by One Way ANOVA, followed by the Tukey post-test. Statistical analyses were performed using GraphPad Prism 9 software. A result was considered significant at p < 0.05.

### Chemical similarity analysis

For all structures, we used SMILES codes as input data for Marvin version 19.27.0 (http://www.chemaxon.com), which included the Standardizer software. This allowed for the conversion of various chemical structures into personalized canonical representations. Standardization is crucial for creating libraries of consistent compounds, as it canonizes the structures, adds hydrogens, aromatizes molecules, generates 3D structures, and saves the compounds in SDF format. Fingerprint descriptors were employed to analyze the similarity between the active molecules. The SDF file was analyzed using AlvaDesc software [17] to calculate the fingerprint descriptors. AlvaDesc can calculate 1024 bits, where each bit encodes the presence or absence of a given fragment. The fingerprints were subjected to hierarchical cluster analysis to classify the 26 molecules, which were then represented through a dendrogram. For similarity analysis, Euclidean distances were utilized, which measure the distance between two points, and which can be demonstrated by repeated application of the Pythagorean theorem. Using this formula as distance, Euclidean space becomes a metric space. The similarity representation was created using a heatmap. Similarity analysis and hierarchical clustering were executed using the Python language and a library called Matplotlib.

### Prediction of absorption, distribution, metabolism, excretion, and toxicity (ADMET) properties

ADMET parameters were calculated using the Swiss ADME open-access web tool [18] (http://www.swissadme.ch)., which offers a set of rapid predictive models for the assessment of physicochemical, pharmacokinetic, and pharmacological properties. The toxicity prediction was performed in the OSIRIS Property Explorer [19] based on the following parameters: mutagenicity, tumorigenicity, reproductive effects and irritability. For absorption, factors included membrane permeability, intestinal absorption and the glycoprotein P substrate or inhibitor. Thus, we investigated compounds that did not exceed more than two violations of the Lipinski rule and for which the logP consensus was not greater than 4.15. In addition, the compounds were not substrates for the permeability glycoprotein enzyme (P-gp). The distribution was evaluated by factors that included the blood-brain barrier (logBB) and the permeability of the CNS. Metabolism was predicted based on the CYP substrate or inhibition models (CYP1A2, CYP2C19, CYP2C9, CYP2D6 and CYP3A4).

## Results

### *In vitro* initial screening

**Fig 1:**
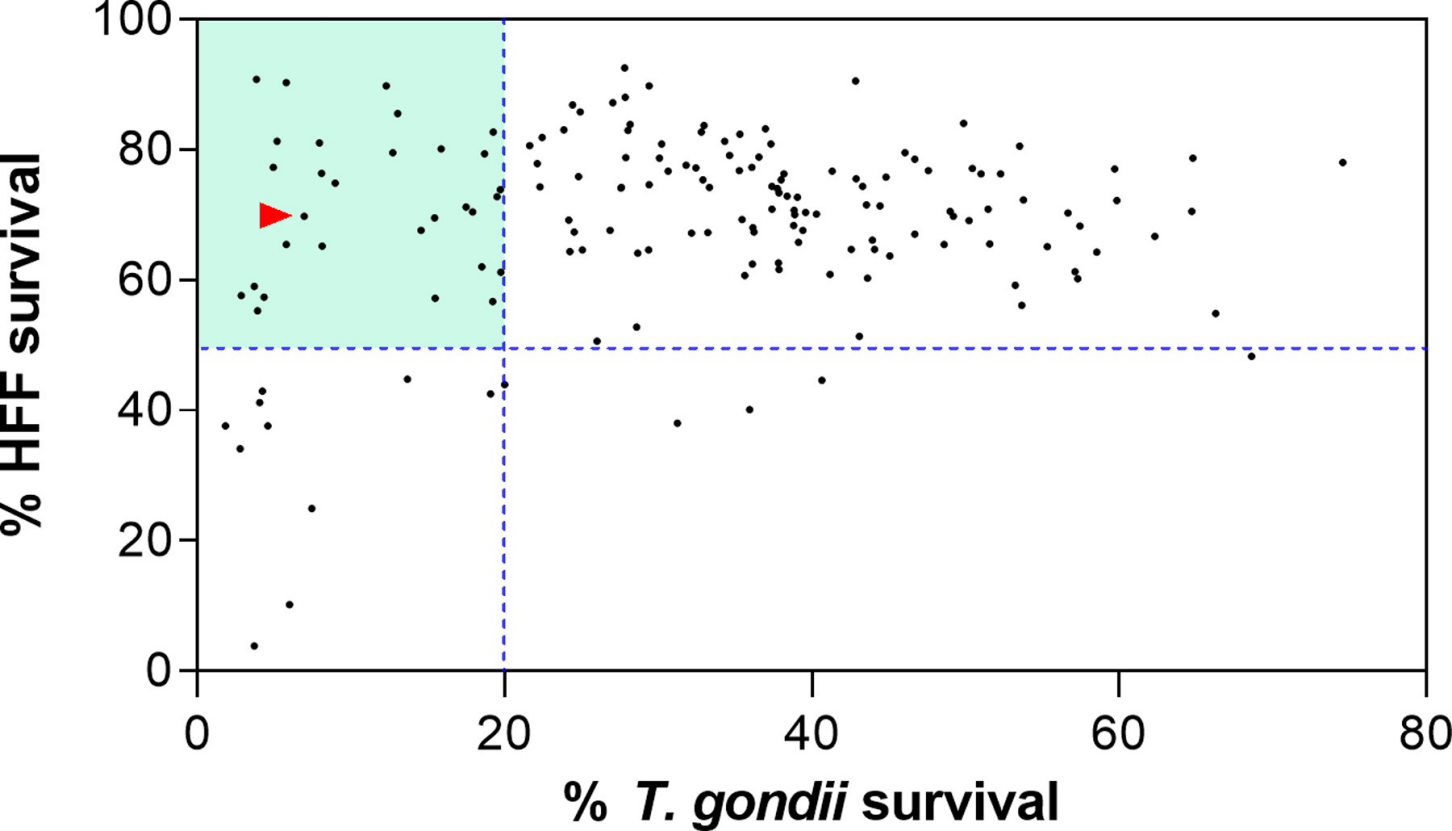
Screening of 160 compounds present in the COVID Box collection against *Toxoplasma gondii* and Human Foreskin Fibroblasts (HFF). Results are presented as the percent survival at 1 μM. The dashed line represents the threshold applied in this study to select compounds with anti-*T*. *gondii* selective activity. The green area shows the selected compounds. The red arrowhead shows the internal control drug pyrimethamine.

The MMV COVID Box collection of 160 compounds was screened against *T*. *gondii* tachyzoites using a β-galactosidase assay and against HFF using the MTT assay to identify the most selective compounds. To do this, we applied a threshold that required compounds to induce at least 80% of inhibition of *T*. *gondii* at 1 uM and less than 50% of inhibition of HFF at the same concentration. This allowed us to identify compounds with submicromolar anti-*T*. *gondii* activity and low cytotoxicity against the host cell. As shown in Fig 1, we identified 29 compounds that inhibited >80% of *T*. *gondii* survival and <50% of HFF survival at 1 μM and selected them for further studies. S1 Fig shows the structures of selected compounds **1** to **29**.

### EC_50_ and CC_50_ determination

The EC_50_ values of the 29 selected compounds against *T*. *gondii* tachyzoites ranged from 0.04 to 0.92 μM, while the CC_50_ values against HFF ranged from 2.48 to >50 μM (the highest tested concentration). The Selectivity Index (SI) of each compound, calculated as the ratio between the CC_50_ and EC_50_, ranged from 19 to >500. Abemaciclib and itraconazole were the most selective compounds (**Table 1**). Pyrimethamine was used as reference drug and the obtained EC_50_ value was consistent with previously reported values [20].

**Table 1:** Selectivity of COVID Box compounds against *T*. *gondii*. EC_50_ against *T*. *gondii* tachyzoites, CC_50_ against HFF and Selectivity Index (SI) of selected compounds, using pyrimethamine as reference drug.

Internal ID	MMV Code	Trivial name	EC_50_ ± SD (μM)^a^	CC_50_ ± SD (μM)^b^	SI^c^	*T*. *gondii* activity^d^
**1**	MMV003461	Niclosamide	0.43 ± 0.15	35.54 ± 9.57	83	[21]
**2**	MMV1804190	Bemcentinib	0.38 ± 0.19	31.55 ± 5.52	83	New
**3**	MMV003140	Ethaverine	0.92 ± 0.09	>50	>54	New
**4**	MMV1804185	Regorafenib	0.40 ± 0.15	11.55 ± 2.19	29	New
**5**	MMV637528	Itraconazole	0.10 ± 0.01	>50	>500	[22]
**6**	MMV662539	Tioguanine	0.46 ± 0.19	>50	>109	New
**7**	MMV690777	LY 2228820	0.23 ± 0.01	26.57 ± 11.55	116	New
**8**	MMV001860	Cyclosporine	0.40 ± 0.10	>50	>125	[23]
**9**	MMV010306	Sorafenib	0.65 ± 0.25	12.40 ± 3.17	19	New
**10**	MMV1804194	Manidipine	0.70 ± 0.25	>50	>71	New
**11**	MMV1804175	Almitrine	0.32 ± 0.14	>50	>156	[24]
**12**	MMV1804174	Abemaciclib	0.05 ± 0.02	>50	>1,000	New
**13**	MMV003277	Tetrandrine	0.49 ± 0.15	34.73 ± 16.98	71	New
**14**	MMV001681	Fluspirilene	0.76 ± 0.07	>50	>66	New
**15**	MMV000068	Tetracycline	0.91 ± 0.09	>50	>55	[25]
**16**	MMV638007	Toremifene	0.85 ± 0.18	44.31 ± 6.80	52	New
**17**	MMV637897	Rapamycin	0.77 ± 0.30	>50	>65	[26]
**18**	MMV007474	Berbamine	0.74 ± 0.34	45.34 ± 5.40	61	New
**19**	MMV1804247	Anidulafungin	0.83 ± 0.21	38.90 ± 15.70	47	New
**20**	MMV1804250	Hanfangchin B	0.84 ± 0.19	44.99 ± 5.85	54	New
**21**	MMV001428	Thiethylperazine	0.90 ± 0.11	33.12 ± 6.01	37	New
**22**	MMV083882	Silmitasertib	0.87 ± 0.15	38.38 ± 16.43	44	New
**23**	MMV1804354	PB 28	0.49 ± 0.21	>50	>102	New
**24**	MMV1804359	Merimepodib	0.78 ± 0.27	26.35 ± 10.00	34	New
**25**	MMV000031	Cycloheximide	0.04 ± 0.02	2.48 ± 0.08	62	[27]
**26**	MMV1804479	AZ3451	0.91 ± 0.09	>50	>55	New
**27**	MMV892669	Desmethyl ferroquine	0.54 ± 0.02	38.22 ± 15.55	71	New
**28**	MMV1804412	Nebivolol	0.78 ± 0.28	37.13 ± 2.88	48	New
**29**	MMV002137	Pimozide	0.82 ± 0.22	>50	>61	[28]
**30**	-	Pyrimetamine	0.37 ± 0.08	>50	>135	-

^a^Half Effective Concentration (EC_50_) against *T*. *gondii* tachyzoites ± Standard Deviation of two independent experiments.

^b^Half Cytotoxic Concentration (CC_50_) against HFF cells ± Standard Deviation of two independent experiments.

^c^Selectivity index (SI), calculated based on the CC_50_ HFF cells/EC_50_ *T*. *gondii* ratio;

^d^In March 2023, we searched on PubMed the trivial name of each compound plus "*Toxoplasma gondii*." If available, the reference for a previous study is included. The term "new" indicates when no results were retrieved from the search.

### ADMET predictions

**Fig 2:**
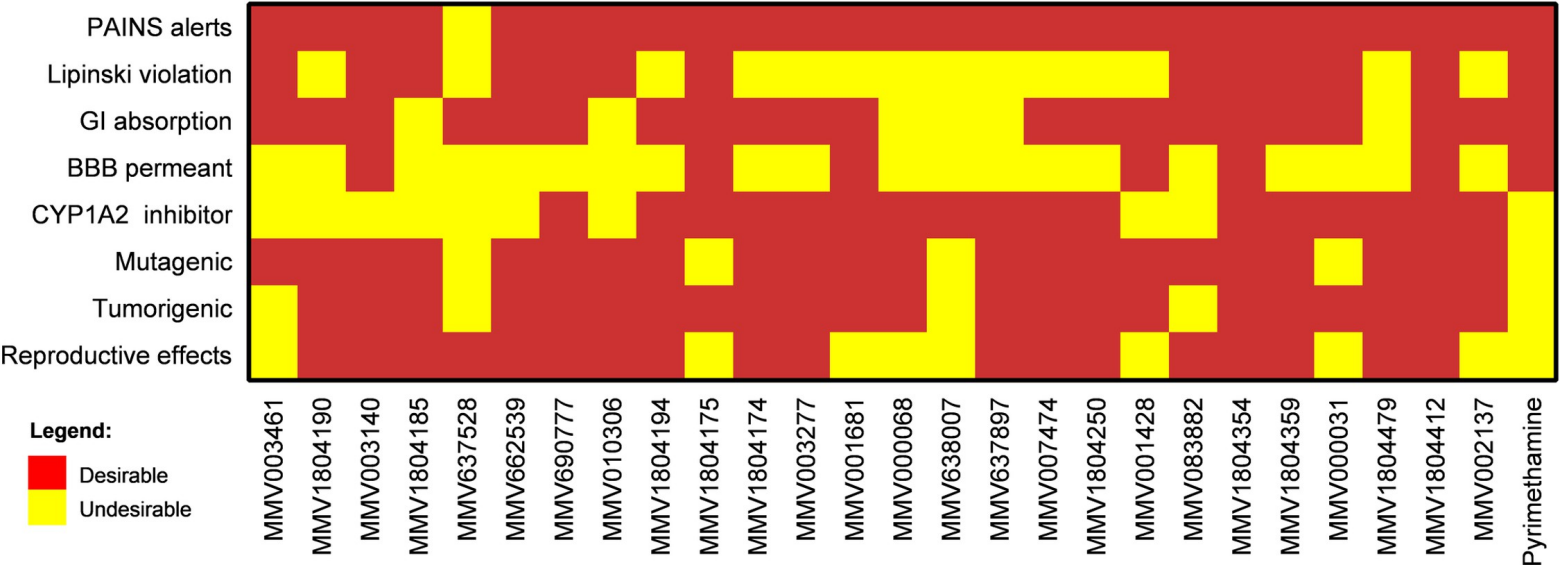
Main results of ADMET parameter analysis for selective anti-*T*. *gondii* compounds from the COVID Box. The figure illustrates the main findings of the ADMET parameter analysis conducted on compounds showing selective *in vitro* activity against *T*. *gondii* from the COVID Box. The objective of the analysis was to identify compounds with favorable pharmacokinetic, pharmacochemical, and pharmacological profiles, thus indicating their potential for further development. Red and yellow colors represent "desirable" and "undesirable" predictions, respectively, indicating the predicted outcomes for each parameter.

The 29 selective compounds were subjected to prediction of several parameters to identify those with the best pharmacokinetic, pharmacochemical, and pharmacological profiles through ADMET properties. Initially, the compounds that have better predictions of oral absorption were verified using Lipinski’s rule of five. Then, the lipophilicity and solubility parameters that contribute to the distribution of the substance *in vivo* were analyzed, which are prerequisites for the molecules to advance in pre-clinical and clinical studies. The parameters that best describe these characteristics are those related to the partition coefficient (LogP). Regarding metabolism, the molecules were analyzed for the probability of being inhibitors of CYP1A2, CYP2C19, CYP2C9, CYP2D6, and CYP3A4 enzymes. The toxicity of these compounds was also analyzed *in silico* for tumorigenicity, mutagenicity, toxic effects on reproductive organs, and dermatological irritants. Fig 2 and supporting information (**S1–S5 Tables**) summarizes the results of the analyzed ADMET parameters. Three compounds could not be analyzed for all ADMET parameters due to the size **(8** and **19**) and complexity of their structure (**27**), which were not supported by the programs used.

Out of the 29 compounds analyzed in terms of medicinal chemistry predictions, it was observed that only compound **5** (itraconazole) exhibited pan-assay interference compounds alerts (PAINS). This indicates that most of the compounds are not predicted to interact non-specifically with multiple biological targets, but rather exhibit reactivity towards one specific desired target.

In terms of druglikeness, it was found that only compounds **2**, **5**, **17**, and **26** had more than two violations of Lipinski’s rule. This suggests that many of the compounds in the study are predicted to have good oral bioavailability.

Physicochemical analysis revealed that 20 compounds exhibited high predicted gastrointestinal absorption, indicating that they have the potential to be effectively absorbed from the gastrointestinal tract. Furthermore, six compounds (**3**, **11**, **14**, **21**, **23**, and **28**) were predicted to have permeation across the blood-brain barrier, suggesting their potential efficacy in treating infections in the central nervous system.

In terms of the interaction with drug-metabolizing enzymes, it was observed that 17 compounds showed no predicted inhibition of CYP1A2, which is a significant human cytochrome P450 enzyme.

Toxicity analysis showed that four compounds (**5**, **11**, **25**, and **27**) were predicted to have high mutagenic properties, four compounds (**1**, **5**, **16**, and **22**) were predicted to have high tumorigenic properties, and seven compounds (**11**, **14**, **15**, **16**, **21**, **25**, and **29**) exhibited a high predicted effect on the reproductive system. Notably, the standard drug pyrimethamine showed high predicted effects on these three parameters (mutagenicity, tumorigenicity, and reproductive).

### Chemical similarity analysis

**Fig 3:**
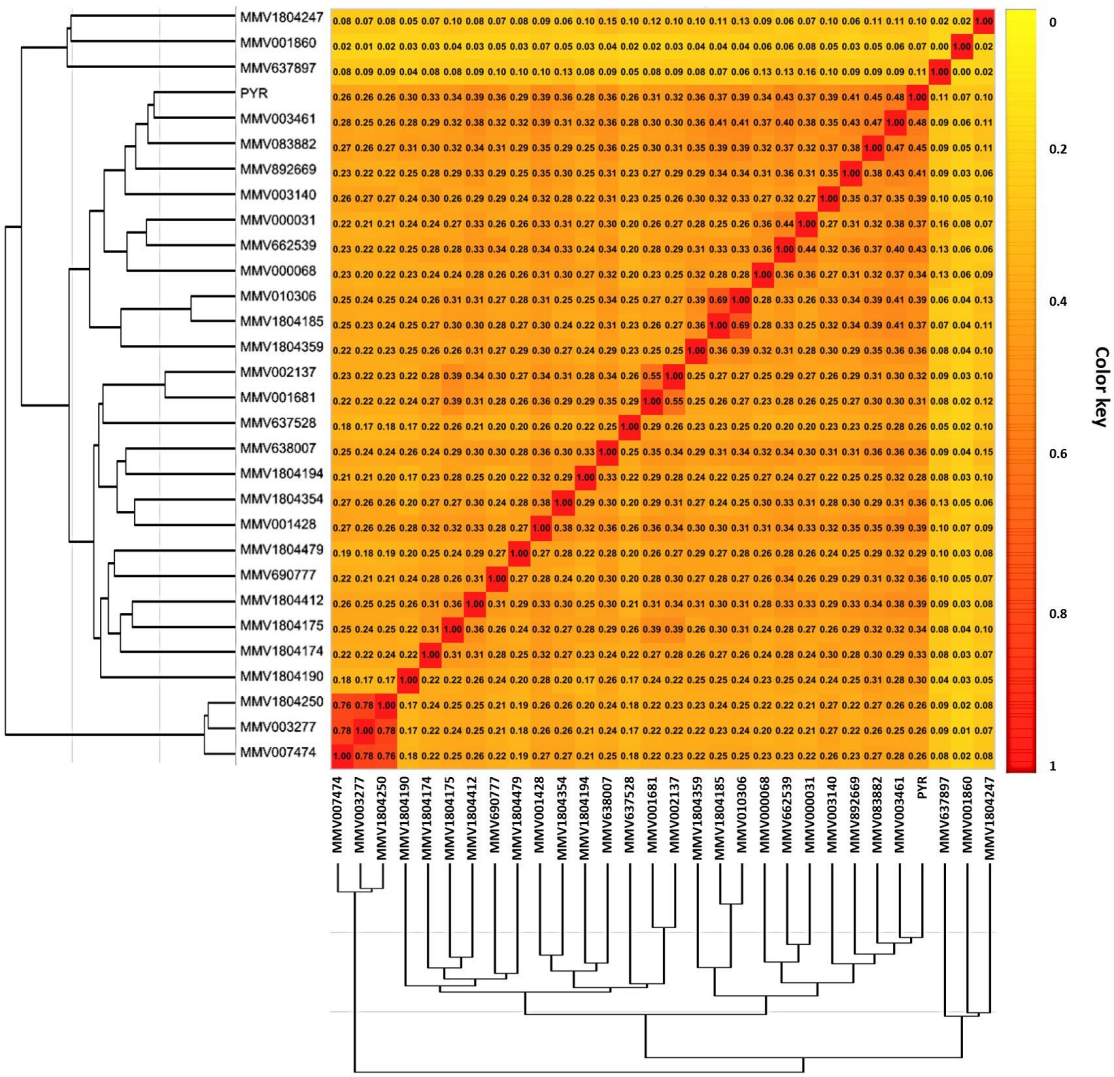
Hierarchical clustering analysis. Structural similarities between the selected compounds and the standard drug Pyrimethamine, according to their Euclidean distance.

This analysis followed the principle of similar properties, which suggests that compounds with similar chemical structures tend to have similar biological properties. In this study, we evaluated 29 compounds with selective activity against *T*. *gondii* for structural similarities among themselves and compared to pyrimethamine (PYR), the positive control used in the *in vitro* tests. Structural similarities were analyzed using Euclidean distances, with smaller distances indicating greater chemical similarity. The heatmap plot expressed this similarity on a scale of 0 to 1, where 0 indicates no similarity and 1 indicates maximum similarity. The clusters obtained through hierarchical cluster analysis (HCA) in Fig 3 highlight the similarities between the 29 selective compounds and PYR. When comparing the 29 active molecules with PYR, we found that the similarity ranged between 0.26 and 0.48, with molecule **1** having the greatest similarity and molecules **5**, **13**, **18**, and **20** having the least similarity with PYR. Comparing the 29 molecules among themselves, the similarity varied between 0.26 and 0.78, with the smallest similarity occurring between molecules **17** and **8** and the greatest similarity between molecules **20** and **13**. It is worth noting that molecules **8**, **17**, and **19** have the least chemical similarity with all molecules, indicating that their structures are quite different from other molecules and even between themselves.

### *In vivo* evaluation of the efficacy of almitrine bismesylate in treating mice chronically infected with *T*. *gondii*

**Fig 4:**
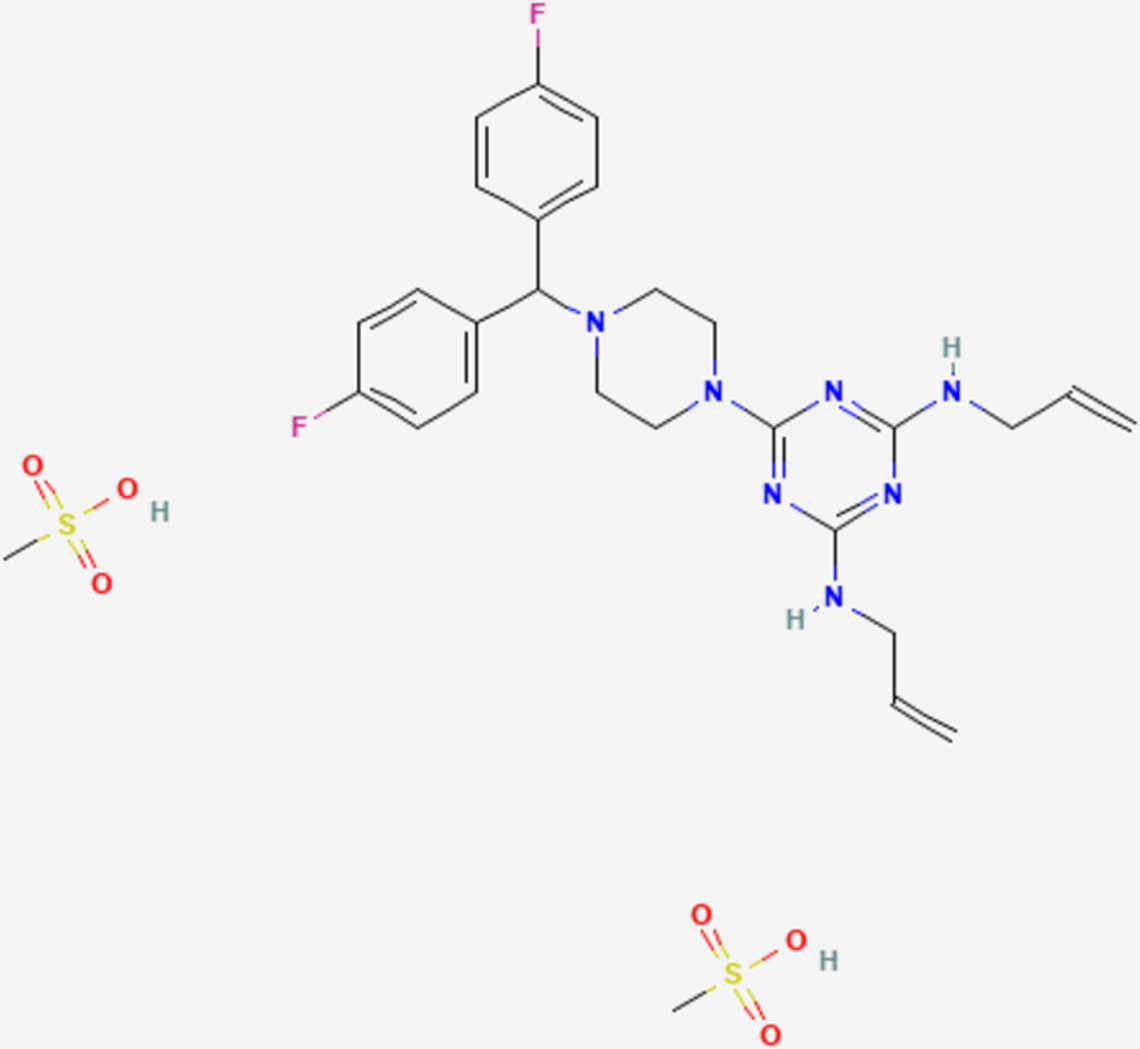
Chemical structure of almitrine bismesylate. Obtained from https://pubchem.ncbi.nlm.nih.gov/.

**Fig 5:**
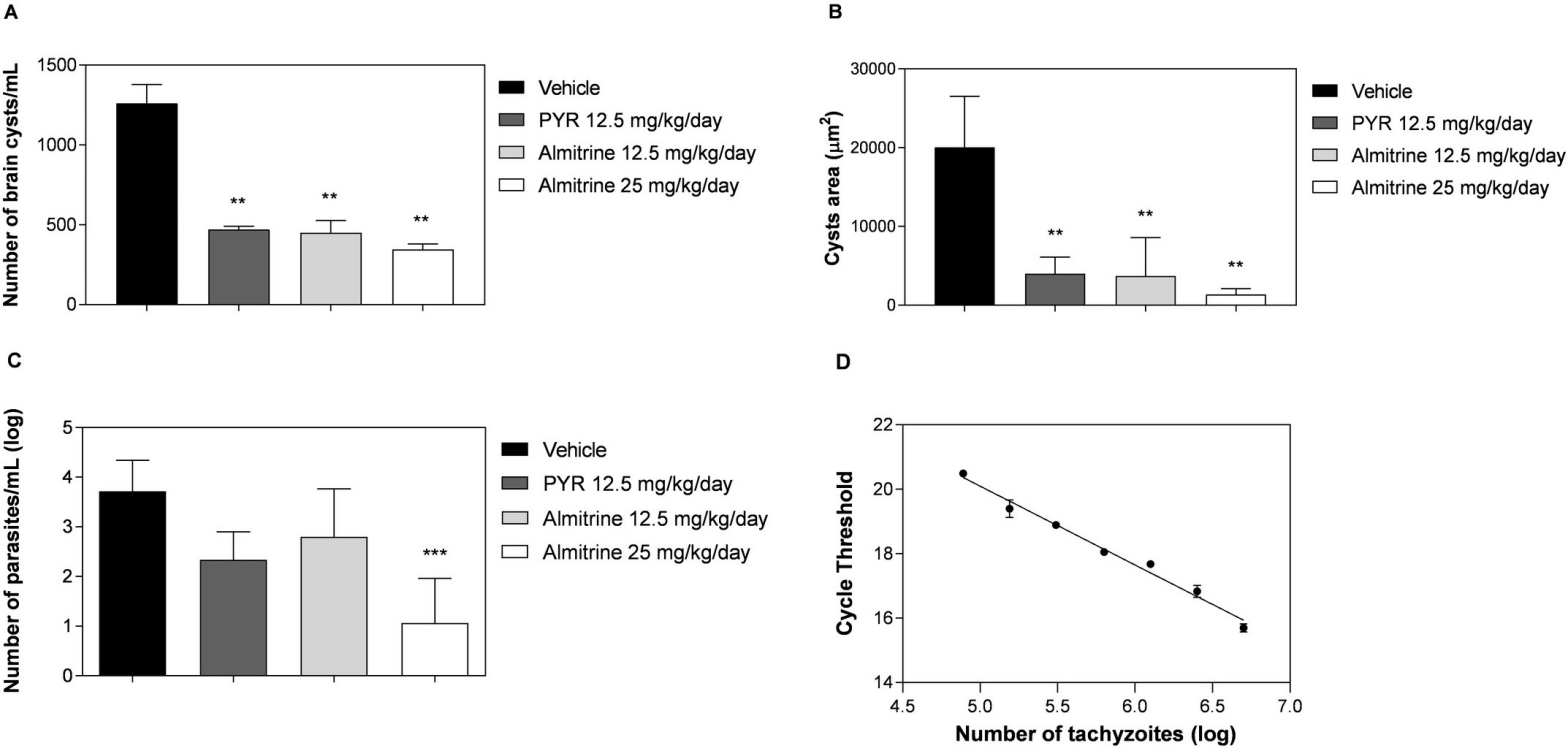
Effect of almitrine bismesylate treatment on parasite burden in mice chronically infected with *T*. *gondii*. Comparative analysis of the number of cysts/mL (A), cysts area (B), and number of parasites/mL (C), performed using a standard curve generated by qPCR (D). Male C57/BL/6 mice (4 weeks-old) were inoculated intraperitoneally with 10 *T*. *gondii* (ME49 strain) cysts. Forty days after infection, mice were randomly assigned into experimental groups (n = 4 per group). These groups received 10 doses of almitrine bismesylate (25 or 12.5 mg/kg/day prepared with DMSO 4% in distilled water) or pyrimethamine (12.5 mg/kg/day prepared with distilled water) by oral gavage in 100 μL final volume. The control group received 100 μL of the vehicle used to dilute almitrine bismesylate (DMSO 4% in distilled water). On day 51 post-infection all animals were euthanized, and their brains were collected for microscopic analysis and RNA quantification. Asterisks indicates significant differences in comparison with the vehicle group.

We evaluated the *in vivo* efficacy of almitrine bismesylate (Fig 4) by administering 12.5 and 25 mg/kg/day doses for 10 consecutive days. Treatment was initiated 40 days after parasite inoculation (ME49 strain), allowing the infection to become chronic. The microscopic observation of brain macerates revealed a significant reduction (p < 0.05) in the number of brain cysts (Fig 5A) and cysts area (Fig 5B) in the treated groups. The real-time PCR quantification results demonstrated a significant (p < 0.001) reduction in parasite burden in the brains of mice treated with almitrine bismesylate at 25 mg/kg/day compared to the vehicle control group (Fig 5C). A standard curve was used to determine the absolute number of parasites, as there is a linear correlation between C_T_ values and parasite number (r^2^ = 0.98) (Fig 5D).

## Discussion

The COVID Box was developed by the MMV in collaboration with scientists from industry and academia, to rapidly respond to the urgent need to combat COVID-19. As previously described, the COVID Box not only supports research against SARS-CoV-2, but also against other human threats [29, 30]. However, this is the first study to evaluate the COVID Box against a human protozoan parasite.

The current study utilizing the COVID Box identified 29 compounds with submicromolar anti-*Toxoplasma* activity and high selectivity indexes. Out of these compounds, eight had previously been identified as anti-*T*. *gondii* agents and served as positive controls, such as itraconazole and tetracycline. Therefore, the present study contributes to the discovery of 21 new anti-*T*. *gondii* drug candidates through screening the COVID Box.

From the 29 compounds selected, 20 exhibited high predicted gastrointestinal absorption, as indicated by the ADME study. Additionally, six compounds were predicted to have the ability to cross the blood-brain barrier, which is a desirable characteristic when considering the chronic phase of toxoplasmosis, wherein the parasite hides in the brain as bradyzoites within cysts. Notably, six novel anti-*T*. *gondii* selective compounds reported here (ethaverine, almitrine, fluspirilene, thuietylperazine, PB38 and nebivolol) are likely to have the chemical and physical properties to be orally bioavailable and to cross the blood-brain barrier.

A great structural diversity among the active molecules was found, a fact that can be observed by the similarity values being mostly below 0.5. Several of the newly described anti-*T*. *gondii* compounds from COVID Box exhibited *in vitro* activity levels similar to that of PYR, despite being structurally distinct from this molecule. As previously reported for the Pathogen Box [8], this discovery could facilitate the development of novel active molecules based on these structures, thereby expanding the range of available therapeutic options.

In a previous study [24], eight compounds from the COVID Box, which had undergone an *in silico* screening based on the proteome of *T*. *gondii*, were *in vitro* evaluated against the parasite. Among these compounds, anti-*T*. *gondii* activity at the nanomolar range, along with selective activity, was observed only for almitrine. In the current study, the entire COVID Box was screened against both HFF cells and *T*. *gondii*, and the *in vivo* efficacy of almitrine bismesylate in *T*. *gondii*-infected mice was further assessed. Therefore, the findings of this study serve to extend and confirm the results of the previous published work.

Out of the 29 selective compounds identified here from the COVID Box, we selected almitrine (MMV1804175) for further *in vivo* studies due to its desirable characteristics, including anti-*T*. *gondii* activity at nanomolar concentrations and low cytotoxicity, resulting in a selectivity index greater than 100. Additionally, almitrine was predicted to have high gastrointestinal absorption and cross the blood-brain barrier, and its *in vivo* efficacy against *T*. *gondii* was unknown. Besides, almitrine showed no predicted inhibition of CYP1A2, suggesting that this compound may have a lower likelihood of causing significant drug-drug interactions or metabolic issues related to CYP1A2 inhibition. Furthermore, the compound’s commercial availability for purchase and approval for clinical use in some countries were also factors considered in our selection.

The administration of almitrine bismesylate (commercially known as Vectarion^®^) via the oral route resulted in a significant reduction in parasite burden in the brains of mice chronically infected with *T*. *gondii*. These findings suggest that almitrine bismesylate may be a promising drug candidate for additional experimental studies on toxoplasmosis. Given its low *in vivo* toxicity (LD_50_ > 200 mg/kg in mice [31]), almitrine bismesylate may be administered at higher doses, which could increase its efficacy against *T*. *gondii*. Furthermore, testing this compound against other experimental models of toxoplasmosis would be worthwhile. Based on a molecular docking previously performed [24] almitrine is a strong candidate to bind the Na^+^/K^+^ ATPase transporter as part of their mechanisms of action. However, further studies are needed to confirm this hypothesis as a possible mechanism of anti-*T*. *gondii* action.

Almitrine bismesylate is a medication commonly prescribed for the treatment of chronic obstructive pulmonary disease (COPD) in humans, typically administered at doses ranging from 50 to 100 mg/day. It is important to note that polyneuropathy is a recognized side effect associated with almitrine use, primarily observed in patients receiving high doses over extended periods of several months. However, these effects are reversible upon discontinuation of treatment. While almitrine does carry risks of toxicity, these risks are significantly minimized when administered at short durations and low doses [32].

We selected a dose of 12.5 mg/kg for the standard drug pyrimethamine, as previously described by [14, 33]. For almitrine bismesylate, we used the same dose as pyrimethamine and included an additional group receiving twice this dose. We based this decision on the low toxicity of almitrine bismesylate at low doses and the allometric scaling from mouse to human.

Considering the Body Surface Area (BSA), the predicted equivalent dose of almitrine bismesylate, based on a mouse dose of 25 mg/kg/day for 10 days, would be approximately 2 mg/kg/day for humans. Therefore, it can be hypothesized that a short-term treatment of 10 days at the low dose of 2 mg/kg would likely result in mild side effects in humans. However, it is crucial to highlight that further research and clinical studies are necessary to determine the safety profile of Vectarion when administered using this treatment regimen.

Regarding the salt form, bismesylate is commonly used as a counterion in pharmaceutical formulations. In general, the salt form of a drug is chosen for its physicochemical properties, stability, solubility, and other factors that can affect the drug’s performance. The salt itself is typically considered inert, meaning it does not have pharmacological activity on its own. However, it’s important to note that we haven’t demonstrated that the salt isn’t active against *T*. *gondii* in mice. Further studies or specific information regarding the use of bismesylate salt in the context of toxoplasmosis treatment would be necessary to provide a comprehensive assessment.

Several compounds have demonstrated efficacy in reducing parasite burden in chronic *T*. *gondii* infection in mice, as reviewed by [34]. However, none of them was able to totally clear the infection in the central nervous system. For instance, atovaquone and ELQ-316 (an endochin-like quinolone), have shown significant reductions in parasite load in *T*. *gondii*-infected mice. Atovaquone at 5 mg/kg reduced the number of brain cysts by 44% and ELQ-316 at 25 mg/kg reduced the number of brain cysts by 88% after 16 days of treatment, as determined by counting the number of brain cysts after the end of the experiment [35].

## Conclusions

The screening of the COVID Box resulted in the identification of new anti-*T*. *gondii* agents, making this the first study to report their activity against this important human parasite. Based on the threshold applied (>80% of inhibition of *T*. *gondii* survival and <50% of inhibition of HFF survival at 1 μM), 29 compounds were found, all of them being at least 10-fold more lethal to the parasite than to the host cell. Based on their highly selective anti-*T*. *gondii* activity and desirable ADME properties, we have identified six compounds that we consider as promising candidates for further preclinical studies on chronic toxoplasmosis. Among them, we selected almitrine for further *in vivo* evaluation and found that almitrine bismesylate was effective in the treatment of mice chronically infected with *T*. *gondii*, resulting in parasite burden reduction in the brain in a dose-dependent manner. The identification of this novel drug candidate holds great relevance, given the lack of effective treatment options available for chronic toxoplasmosis. The results presented here provide further evidence of the potential of the MMV collections as a valuable source of drugs to be repositioned for infectious diseases.

## Supporting information

S1 Table Physicochemical properties predictions. (PDF)

S2 Table Lipophilicity and toxicity predictions. (PDF)

S3 Table Water solubility predictions. (PDF)

S4 Table Physicochemical properties predictions. (PDF)

S5 Table Drug likeness and medicinal chemistry predictions. (PDF)

S1 Fig Structures of selected compounds 1 to 29. (PDF)

## References

[1] Hill DE, Chirukandoth S, Dubey JP. Biology and epidemiology of *Toxoplasma gondii* in man and animals. Anim Heal Res Rev. 2005;6: 41–61. doi: 10.1079/AHR2005100 16164008

[2] Flegr J, Prandota J, Sovičková M, Israili ZH. Toxoplasmosis–A Global Threat. Correlation of Latent Toxoplasmosis with Specific Disease Burden in a Set of 88 Countries. PLoS One. 2014;9: e90203. doi: 10.1371/journal.pone.0090203 24662942 PMC3963851

[3] Montazeri M, Sharif M, Sarvi S, Mehrzadi S, Ahmadpour E, Daryani A. A systematic review of in vitro and in vivo activities of anti-toxoplasma drugs and compounds (2006–2016). Front Microbiol. 2017;8. doi: 10.3389/fmicb.2017.00025 28163699 PMC5247447

[4] Pushpakom S, Iorio F, Eyers PA, Escott KJ, Hopper S, Wells A, et al. Drug repurposing: progress, challenges and recommendations. Nat Rev Drug Discov. 2019;18: 41–58. doi: 10.1038/nrd.2018.168 30310233

[5] Andrews KT, Fisher G, Skinner-Adams TS. Drug repurposing and human parasitic protozoan diseases. Int J Parasitol Drugs drug Resist. 2014;4: 95–111. doi: 10.1016/j.ijpddr.2014.02.002 25057459 PMC4095053

[6] Hajj R El, Tawk L, Itani S, Hamie M, Ezzeddine J, Sabban M El, et al. Toxoplasmosis: Current and Emerging Parasite Druggable Targets. Microorganisms. 2021;9. doi: 10.3390/MICROORGANISMS9122531 34946133 PMC8707595

[7] Boyom FF, Fokou PVT, Tchokouaha LRY, Spangenberg T, Mfopa AN, Kouipou RMT, et al. Repurposing the open access malaria box to discover potent inhibitors of Toxoplasma gondii and Entamoeba histolytica. Antimicrob Agents Chemother. 2014;58: 5848–5854. doi: 10.1128/AAC.02541-14 25049259 PMC4187973

[8] Spalenka J, Escotte-Binet S, Bakiri A, Hubert J, Renault J-H, Velard F, et al. Discovery of New Inhibitors of Toxoplasma gondii via the Pathogen Box. Antimicrob Agents Chemother. 2018;62. doi: 10.1128/AAC.01640-17 29133550 PMC5786798

[9] Castro AS, Alves CMOS, Angeloni MB, Gomes AO, Barbosa BF, Franco PS, et al. Trophoblast cells are able to regulate monocyte activity to control Toxoplasma gondii infection. Placenta. 2013;34: 240–247. doi: 10.1016/j.placenta.2012.12.006 23294571

[10] Wang Q, Sibley LD. Assays for Monitoring Toxoplasma gondii Infectivity in the Laboratory Mouse. Methods Mol Biol. 2020;2071: 99–116. doi: 10.1007/978-1-4939-9857-9_5 31758448

[11] Chin FT, Xing WZ, Bogyo M, Carruthers VB. Cysteine protease inhibitors block Toxoplasma gondii microneme secretion and cell invasion. Antimicrob Agents Chemother. 2007;51: 679–688. doi: 10.1128/AAC.01059-06 17145790 PMC1797762

[12] Galal KA, Truong A, Kwarcinski F, de Silva C, Avalani K, Havener TM, Chirgwin ME, Merten E, Ong HW, Willis C, Abdelwaly A, Helal MA, Derbyshire ER, Zutshi R, Drewry DH. Identification of Novel 2,4,5-Trisubstituted Pyrimidines as Potent Dual Inhibitors of Plasmodial PfGSK3/PfPK6 with Activity against Blood Stage Parasites In Vitro. J Med Chem. 2022; 13;65(19): 13172–13197. doi: 10.1021/acs.jmedchem.2c00996 36166733 PMC9574854

[13] Reimão JQ, Mesquita JT, Ferreira DD, Tempone AG. Investigation of Calcium Channel Blockers as Antiprotozoal Agents and Their Interference in the Metabolism of Leishmania (L.) infantum. Evid Based Complement Alternat Med. 2016;2016. doi: 10.1155/2016/1523691 26941821 PMC4749844

[14] Nishi L, Santana PL, Evangelista FF, Beletini LF, Souza AH, Mantelo FM, et al. Rosuvastatin reduced brain parasite burden in a chronic toxoplasmosis in vivo model and influenced the neuropathological pattern of ME-49 strain. Parasitology. 2020;147: 303–309. doi: 10.1017/S0031182019001604 31727196 PMC10317618

[15] Reimão JQ, Colombo FA, Pereira-Chioccola VL, Tempone AG. In vitro and experimental therapeutic studies of the calcium channel blocker bepridil: Detection of viable Leishmania (L.) chagasi by real-time PCR. Exp Parasitol. 2011;128: 111–115. doi: 10.1016/j.exppara.2011.02.021 21354141

[16] Pomares C, Estran R, Press CJ, Bera A, Ramirez R, Montoya JG, et al. Is real-time PCR targeting rep 529 suitable for diagnosis of toxoplasmosis in patients infected with non-type II strains in North America? J Clin Microbiol. 2020;58: 12–16. doi: 10.1128/JCM.01223-19 31694976 PMC6989067

[17] Mauri A. alvaDesc: A tool to calculate and analyze molecular descriptors and fingerprints. Methods Pharmacol Toxicol. 2020; 801–820. doi: 10.1007/978-1-0716-0150-1_32/COVER

[18] Daina A, Michielin O, Zoete V. SwissADME: a free web tool to evaluate pharmacokinetics, drug-likeness and medicinal chemistry friendliness of small molecules. Sci Reports 2017 71. 2017;7: 1–13. doi: 10.1038/srep42717 28256516 PMC5335600

[19] Sander T, Freyss J, Von Korff M, Reich JR, Rufener C. OSIRIS, an entirely in-house developed drug discovery informatics system. J Chem Inf Model. 2009;49: 232–246. doi: 10.1021/ci800305f 19434825

[20] Radke JB, Burrows JN, Goldberg DE, Sibley LD. Evaluation of Current and Emerging Antimalarial Medicines for Inhibition of Toxoplasma gondii Growth in Vitro. ACS Infect Dis. 2018;4: 1264–1274. doi: 10.1021/acsinfecdis.8b00113 29998728 PMC6093624

[21] Zhang JLY, Si HF, Shang XF, Zhang XK, Li B, Zhou XZ, et al. New life for an old drug: In vitro and in vivo effects of the anthelmintic drug niclosamide against Toxoplasma gondii RH strain. Int J Parasitol Drugs drug Resist. 2019;9: 27–34. doi: 10.1016/j.ijpddr.2018.12.004 30599391 PMC6312869

[22] Martins-Duarte ÉS, Lemgruber L, de Souza W, Vommaro RC. Toxoplasma gondii: Fluconazole and itraconazole activity against toxoplasmosis in a murine model. Exp Parasitol. 2010;124: 466–469. doi: 10.1016/j.exppara.2009.12.011 20045696

[23] Chappell LH, Wastling JM. Cyclosporin A: antiparasite drug, modulator of the host-parasite relationship and immunosuppressant. Parasitology. 1992;105 Suppl: S25–S40. doi: 10.1017/s0031182000075338 1308927

[24] Cajazeiro DC, Toledo PPM, de Sousa NF, Scotti MT, Reimão JQ. Drug Repurposing Based on Protozoan Proteome: In Vitro Evaluation of In Silico Screened Compounds against Toxoplasma gondii. Pharmaceutics. 2022;14. doi: 10.3390/pharmaceutics14081634 36015260 PMC9414507

[25] Tibble MJK, Selwyn S. Tetracycline treatment in a food-borne outbreak of toxoplasmosis. Br Med J. 1977;1: 1064. doi: 10.1136/BMJ.1.6068.1064 858046 PMC1606079

[26] Souto XM, Barbosa HS, Menna-Barreto RFS. The morphological analysis of autophagy in primary skeletal muscle cells infected with Toxoplasma gondii. Parasitol Res. 2016;115: 2853–2861. doi: 10.1007/s00436-016-5040-3 27075305

[27] Beckers CJM, Roos DS, Donald RGK, Luft BJ, Schwab JC, Cao Y, et al. Inhibition of cytoplasmic and organellar protein synthesis in Toxoplasma gondii. Implications for the target of macrolide antibiotics. J Clin Invest. 1995;95: 367–376. doi: 10.1172/JCI117665 7814637 PMC295440

[28] Dittmar AJ, Drozda AA, Blader IJ. Drug Repurposing Screening Identifies Novel Compounds That Effectively Inhibit Toxoplasma gondii Growth. mSphere. 2016;1. doi: 10.1128/mSphere.00042-15 27303726 PMC4894684

[29] Almeida-Paes R, de Andrade IB, Ramos MLM, Rodrigues MV de A, Do Nascimento VA, Bernardes-Engemann AR, et al. Medicines for Malaria Venture COVID Box: a source for repurposing drugs with antifungal activity against human pathogenic fungi. Mem Inst Oswaldo Cruz. 2021;116. doi: 10.1590/0074-02760210207 34755820 PMC8577065

[30] Vanmechelen B, Stroobants J, Chiu W, Schepers J, Marchand A, Chaltin P, et al. Identification of novel Ebola virus inhibitors using biologically contained virus. Antiviral Res. 2022;200. doi: 10.1016/j.antiviral.2022.105294 35337896

[31] Golder FJ, Hewitt MM, McLeod JF. Respiratory stimulant drugs in the post-operative setting. Respir Physiol Neurobiol. 2013;189: 395–402. doi: 10.1016/j.resp.2013.06.010 23791825

[32] Silva-Costa-Gomes T, Gallart L, Vallès J, Trillo L, Minguella J, Puig MM. Low- vs high-dose almitrine combined with nitric oxide to prevent hypoxia during open-chest one-lung ventilation. Br J Anaesth. 2005; 95(3): 410–6. doi: 10.1093/bja/aei194 16024585

[33] Silva LA, Fernandes MD, Machado AS, Reis-Cunha JL, Bartholomeu DC, Almeida Vitor RW. Efficacy of sulfadiazine and pyrimetamine for treatment of experimental toxoplasmosis with strains obtained from human cases of congenital disease in Brazil. Exp Parasitol. 2019; 202: 7–14. doi: 10.1016/j.exppara.2019.05.001 31077733

[34] Montazeri M, Mehrzadi S, Sharif M, Sarvi S, Shahdin S, Daryani A. Activities of anti-Toxoplasma drugs and compounds against tissue cysts in the last three decades (1987 to 2017), a systematic review. Parasitol Res. 2018;117(10): 3045–3057. doi: 10.1007/s00436-018-6027-z 30088074

[35] Doggett JS, Nilsen A, Forquer I, Wegmann KW, Jones-Brando L, Yolken RH, Bordón C, Charman SA, Katneni K, Schultz T, Burrows JN, Hinrichs DJ, Meunier B, Carruthers VB, Riscoe MK. Endochin-like quinolones are highly efficacious against acute and latent experimental toxoplasmosis. Proc Natl Acad Sci U S A. 2012;109(39): 15936–41. doi: 10.1073/pnas.1208069109 23019377 PMC3465437

