# Supplementary material for: Repurposing the Medicines for Malaria Venture’s COVID Box to discover potent inhibitors of Toxoplasma gondii, and in vivo efficacy evaluation of almitrine bismesylate (MMV1804175) in chronically infected mice
Source: PLoS One. 2023 Jul 7;18(7):e0288335. doi: 10.1371/journal.pone.0288335 (PMC10328330; doi:10.1371/journal.pone.0288335)
Supplement: S1 Table — (PDF) [file pone.0288335.s001.pdf]

MMV Code	ID	Physicochemical properties									
		Molecular Formula	Molecular Weight	Heavy atoms	Aromatic heavy atoms	Fraction Csp3	Rotatable bonds	H-bond acceptors	H-bond donors	Molar Refractivity	TPSA[2]
MMV003461	1	$C_{13}H_8Cl_2N_2O_4$	327.12	21	12	0	4	4	2	81.52	95.15
MMV1804190	2	$C_{30}H_{34}N_8$	506.64	38	23	0.4	4	5	2	155.12	97.78
MMV003140	3	$C_{24}H_{29}NO_4$	395.49	29	16	0.38	10	5	0	116.39	49.81
MMV1804185	4	$C_{21}H_{15}ClF_4N_4O_3$	482.82	33	18	0.1	9	8	3	112.44	92.35
MMV637528	5	$C_{35}H_{38}Cl_2N_8O_4$	705.63	49	28	0.37	11	7	0	194.53	104.7
MMV662539	6	$C_5H_5N_5S$	167.19	11	9	0	0	2	3	43.48	115.47
MMV690777	7	$C_{24}H_{29}FN_6$	420.53	31	20	0.38	5	4	2	124.41	85.41
MMV001860	8	-	-	-	-	-	-	-	-	-	-
MMV010306	9	$C_{21}H_{16}ClF_3N_4O_3$	464.82	32	18	0.1	9	7	3	112.48	92.35
MMV1804194	10	$C_{35}H_{38}N_4O_6$	610.7	45	18	0.31	12	8	1	183.85	116.93
MMV1804175	11	$C_{26}H_{29}F_2N_7$	477.55	35	18	0.27	10	6	2	141.87	69.21
MMV1804174	12	$C_{27}H_{32}F_2N_8$	506.59	37	21	0.41	7	8	1	149.17	75
MMV003277	13	$C_{38}H_{42}N_2O_6$	622.75	46	24	0.37	4	8	0	186.07	61.86
MMV001681	14	$C_{29}H_{31}F_2N_3O$	475.57	35	18	0.34	7	4	1	145.27	35.58
MMV000068	15	$C_{22}H_{24}N_2O_8$	444.43	32	6	0.41	2	9	6	110.79	181.62
MMV638007	16	$C_{26}H_{28}ClNO$	405.96	29	18	0.23	9	2	0	124.52	12.47
MMV637897	17	$C_{51}H_{79}NO_{13}$	914.17	65	0	0.75	6	13	3	253.68	195.43
MMV007474	18	$C_{37}H_{40}N_2O_6$	608.72	45	24	0.35	3	8	1	181.6	72.86
MMV1804247	19	-	-	-	-	-	-	-	-	-	-
MMV1804250	20	$C_{37}H_{40}N_2O_6$	608.72	45	24	0.35	3	8	1	181.6	72.86
MMV001428	21	$C_{22}H_{29}N_3S_2$	399.62	27	12	0.45	6	2	0	129.63	60.32
MMV083882	22	$C_{19}H_{12}ClN_3O_2$	349.77	25	20	0	3	4	2	98.56	75.11
MMV1804354	23	$C_{24}H_{38}N_2O$	370.57	27	6	0.75	6	3	0	122.25	15.71
MMV1804359	24	$C_{23}H_{24}N_4O_6$	452.46	33	17	0.26	11	7	3	119.6	123.95
MMV000031	25	$C_{15}H_{23}NO_4$	281.35	20	0	0.8	3	4	2	78.47	83.47
MMV1804479	26	$C_{30}H_{27}BrN_4O_3$	571.46	38	21	0.3	6	5	1	150.66	89.17
MMV892669	27	-	-	-	-	-	-	-	-	-	-
MMV1804412	28	$C_{22}H_{25}F_2NO_4$	405.44	29	12	0.45	6	7	3	103.34	70.95
MMV002137	29	$C_{28}H_{29}F_2N_3O$	461.55	34	21	0.32	7	4	1	135.86	41.03
Pyrimethamine[1]	30	$C_{13}H_8Cl_2N_2O_4$	248.71	17	12	0.17	2	2	2	71.06	77.82

[1]Positive control; [2]Total polar surface area.
