# Supplementary material for: Repurposing the Medicines for Malaria Venture’s COVID Box to discover potent inhibitors of Toxoplasma gondii, and in vivo efficacy evaluation of almitrine bismesylate (MMV1804175) in chronically infected mice
Source: PLoS One. 2023 Jul 7;18(7):e0288335. doi: 10.1371/journal.pone.0288335 (PMC10328330; doi:10.1371/journal.pone.0288335)
Supplement: S2 Table — (PDF) [file pone.0288335.s002.pdf]

MMV Code	ID	Lipophilicity						Toxicity			
		iLOGP	XLOGP3	WLOGP	MLOGP	Silicos-IT Log P	Consensus Log P	Mutagenic	Tumorigenic	Reproductive effects	Irritant
MMV003461	1	2.09	5.32	3.67	2.44	1.22	2.95	none	high	low	low
MMV1804190	2	4.51	5.55	4.51	4.48	3.58	4.53	none	none	none	none
MMV003140	3	4.47	4.42	5.42	2.65	6.11	4.61	none	none	none	none
MMV1804185	4	3.51	4.17	6.88	3.28	4.21	4.41	none	none	none	none
MMV637528	5	5.26	5.66	4.71	4.21	3.7	4.71	high	high	none	none
MMV662539	6	0.14	-0.07	0.61	-1.31	2.05	0.28	none	none	none	none
MMV690777	7	3.79	5.32	5.98	3.01	5.17	4.65	none	none	none	none
MMV001860	8	-	-	-	-	-	-	none	none	none	none
MMV010306	9	3.42	4.07	6.32	2.91	3.78	4.1	none	none	none	none
MMV1804194	10	5.09	5.2	3.49	2.54	3.15	3.89	none	none	none	none
MMV1804175	11	4.86	5.55	3.63	3.77	4.32	4.42	high	none	high	high
MMV1804174	12	4.16	3.84	4.86	2.63	4.28	3.96	none	none	none	none
MMV003277	13	5.23	6.66	5.75	3.73	6.06	5.49	none	none	none	none
MMV001681	14	4.16	5.86	5	5.11	5.8	5.19	none	none	high	none
MMV000068	15	0.54	0.26	-0.48	-2.08	-0.59	-0.47	none	none	high	none
MMV638007	16	4.7	7.2	6.22	5.3	6.5	5.98	low	high	high	none
MMV637897	17	6.33	6.02	5.8	1	3.66	4.56	none	none	none	none
MMV007474	18	4.82	6.34	5.45	3.55	5.5	5.13	none	none	none	none
MMV1804247	19	-	-	-	-	-	-	none	none	none	none
MMV1804250	20	4.88	6.34	5.45	3.55	5.5	5.14	none	none	none	none
MMV001428	21	4.29	5.41	3.9	4.37	4.21	4.43	none	none	high	none
MMV083882	22	2.11	4.4	4.88	2.75	3.69	3.57	none	high	none	none
MMV1804354	23	4.64	5.44	4.08	3.98	4.78	4.58	none	none	none	none
MMV1804359	24	3.15	2.1	3.48	0.92	2.15	2.36	none	none	none	none
MMV000031	25	1.83	0.55	0.66	1.19	1.92	1.23	high	none	high	high
MMV1804479	26	4.21	6.86	7.27	4.22	5.93	5.7	none	none	none	none
MMV892669	27	-	-	-	-	-	-	high	none	none	none
MMV1804412	28	3.89	4.04	3.2	2.64	4.14	3.58	none	none	none	none
MMV002137	29	4.23	6.3	6.32	5.45	6.33	5.72	none	none	high	none
Pyrimethamine[1]	30	2.15	2.69	2.54	1.64	2.44	2.29	high	high	high	none

[1]Positive control.
