# Supplementary material for: Repurposing the Medicines for Malaria Venture’s COVID Box to discover potent inhibitors of Toxoplasma gondii, and in vivo efficacy evaluation of almitrine bismesylate (MMV1804175) in chronically infected mice
Source: PLoS One. 2023 Jul 7;18(7):e0288335. doi: 10.1371/journal.pone.0288335 (PMC10328330; doi:10.1371/journal.pone.0288335)
Supplement: S3 Table — (PDF) [file pone.0288335.s003.pdf]

| MMV Code | ID | Water solubility | | | | | | | | | | | |
|---|---|---|---|---|---|---|---|---|---|---|---|---|---|
| | | ESOL Log S | ESOL Solubility (mg/ml) | ESOL Solubility (mol/l) | ESOL Class | Ali Log S | Ali Solubility (mg/ml) | Ali Solubility (mol/l) | Ali Class | Silicos-IT LogSw | Silicos-IT Solubility (mg/ml) | Silicos-IT Solubility (mol/l) | Silicos-IT class |
| MMV003461 | 1 | -5.38 | 1.37E-03 | 4.18E-06 | MS[2] | -7.07 | 2.78E-05 | 8.51E-08 | PS | -4.93 | 3.87E-03 | 1.18E-05 | MS |
| MMV1804190 | 2 | -6.66 | 1.10E-04 | 2.18E-07 | PS[3] | -7.36 | 2.19E-05 | 4.33E-08 | PS | -8.78 | 8.35E-07 | 1.65E-09 | PS |
| MMV003140 | 3 | -4.82 | 5.92E-03 | 1.50E-05 | MS | -5.18 | 2.59E-03 | 6.55E-06 | MS | -8.68 | 8.30E-07 | 2.10E-09 | PS |
| MMV1804185 | 4 | -5.27 | 2.59E-03 | 5.37E-06 | MS | -5.82 | 7.35E-04 | 1.52E-06 | MS | -8.86 | 6.63E-07 | 1.37E-09 | PS |
| MMV637528 | 5 | -7.48 | 2.35E-05 | 3.33E-08 | PS | -7.62 | 1.68E-05 | 2.38E-08 | PS | -9.24 | 4.10E-07 | 5.81E-10 | PS |
| MMV662539 | 6 | -1.44 | 6.10E+00 | 3.65E-02 | VS[4] | -1.9 | 2.09E+00 | 1.25E-02 | VS | -1.91 | 2.06E+00 | 1.23E-02 | S |
| MMV690777 | 7 | -5.95 | 4.76E-04 | 1.13E-06 | MS | -6.87 | 5.73E-05 | 1.36E-07 | PS | -8.23 | 2.45E-06 | 5.82E-09 | PS |
| MMV001860 | 8 | - | - | - | - | - | - | - | - | - | - | - | - |
| MMV010306 | 9 | -5.11 | 3.62E-03 | 7.79E-06 | MS | -5.71 | 8.98E-04 | 1.93E-06 | MS | -8.6 | 1.16E-06 | 2.50E-09 | PS |
| MMV1804194 | 10 | -6.41 | 2.40E-04 | 3.92E-07 | PS | -7.4 | 2.42E-05 | 3.96E-08 | PS | -8.82 | 9.18E-07 | 1.50E-09 | PS |
| MMV1804175 | 11 | -6.02 | 4.58E-04 | 9.60E-07 | PS | -6.76 | 8.23E-05 | 1.72E-07 | PS | -8.36 | 2.11E-06 | 4.42E-09 | PS |
| MMV1804174 | 12 | -5.36 | 2.22E-03 | 4.38E-06 | MS | -5.11 | 3.92E-03 | 7.75E-06 | MS | -8.62 | 1.23E-06 | 2.42E-09 | PS |
| MMV003277 | 13 | -8.02 | 5.96E-06 | 9.57E-09 | PS | -7.76 | 1.08E-05 | 1.73E-08 | PS | -10.8 | 9.78E-09 | 1.57E-11 | IS[6] |
| MMV001681 | 14 | -6.4 | 1.90E-04 | 3.99E-07 | PS | -6.38 | 1.99E-04 | 4.18E-07 | PS | -9.64 | 1.08E-07 | 2.28E-10 | PS |
| MMV000068 | 15 | -2.77 | 7.62E-01 | 1.71E-03 | S[5] | -3.64 | 1.03E-01 | 2.32E-04 | S | -1.82 | 6.68E+00 | 1.50E-02 | S |
| MMV638007 | 16 | -6.76 | 7.08E-05 | 1.74E-07 | PS | -7.28 | 2.11E-05 | 5.19E-08 | PS | -9.52 | 1.21E-07 | 2.99E-10 | PS |
| MMV637897 | 17 | -8.9 | 1.14E-06 | 1.25E-09 | PS | -9.9 | 1.15E-07 | 1.25E-10 | PS | -5.58 | 2.38E-03 | 2.61E-06 | MS |
| MMV007474 | 18 | -7.8 | 9.54E-06 | 1.57E-08 | PS | -7.66 | 1.33E-05 | 2.19E-08 | PS | -10.12 | 4.61E-08 | 7.57E-11 | IS |
| MMV1804247 | 19 | - | - | - | - | - | - | - | - | - | - | - | - |
| MMV1804250 | 20 | -7.8 | 9.54E-06 | 1.57E-08 | PS | -7.66 | 1.33E-05 | 2.19E-08 | PS | -10.12 | 4.61E-08 | 7.57E-11 | IS |
| MMV001428 | 21 | -5.66 | 8.77E-04 | 2.19E-06 | MS | -6.43 | 1.48E-04 | 3.70E-07 | PS | -6.56 | 1.10E-04 | 2.74E-07 | PS |
| MMV083882 | 22 | -5.17 | 2.34E-03 | 6.69E-06 | MS | -5.69 | 7.07E-04 | 2.02E-06 | MS | -7.52 | 1.05E-05 | 3.00E-08 | PS |
| MMV1804354 | 23 | -5.33 | 1.72E-03 | 4.64E-06 | MS | -5.53 | 1.10E-03 | 2.98E-06 | MS | -5.78 | 6.14E-04 | 1.66E-06 | MS |
| MMV1804359 | 24 | -3.62 | 1.08E-01 | 2.38E-04 | S | -4.33 | 2.10E-02 | 4.64E-05 | MS | -7.09 | 3.68E-05 | 8.13E-08 | PS |
| MMV000031 | 25 | -1.73 | 5.20E+00 | 1.85E-02 | VS | -1.87 | 3.75E+00 | 1.33E-02 | VS | -2.38 | 1.17E+00 | 4.16E-03 | S |
| MMV1804479 | 26 | -7.72 | 1.09E-05 | 1.92E-08 | PS | -8.54 | 1.64E-06 | 2.87E-09 | PS | -9.42 | 2.18E-07 | 3.82E-10 | PS |
| MMV892669 | 27 | - | - | - | - | - | - | - | - | - | - | - | - |
| MMV1804412 | 28 | -4.81 | 6.29E-03 | 1.55E-05 | MS | -5.23 | 2.37E-03 | 5.84E-06 | MS | -5.88 | 5.37E-04 | 1.32E-06 | MS |
| MMV002137 | 29 | -6.67 | 9.97E-05 | 2.16E-07 | PS | -6.95 | 5.17E-05 | 1.12E-07 | PS | -9.21 | 2.88E-07 | 6.24E-10 | PS |
| Pyrimethamine[1] | 30 | -3.47 | 8.48E-02 | 3.41E-04 | S | -3.98 | 2.62E-02 | 1.05E-04 | S | -4.87 | 3.39E-03 | 1.36E-05 | MS |

[1]Positive control; [2]MS: Moderately soluble; [3]PS: Poorly soluble; [4]VS: Very soluble [5]S: Soluble; [6]IS: Insoluble.
