# Supplementary material for: Repurposing the Medicines for Malaria Venture’s COVID Box to discover potent inhibitors of Toxoplasma gondii, and in vivo efficacy evaluation of almitrine bismesylate (MMV1804175) in chronically infected mice
Source: PLoS One. 2023 Jul 7;18(7):e0288335. doi: 10.1371/journal.pone.0288335 (PMC10328330; doi:10.1371/journal.pone.0288335)
Supplement: S4 Table — (PDF) [file pone.0288335.s004.pdf]

MMV Code	ID	Physicochemical properties								
		GI[2] absorption	BBB[3] permeant	Pgp[3] substrate	CYP1A2 inhibitor	CYP2C19 inhibitor	CYP2C9 inhibitor	CYP2D6 inhibitor	CYP3A4 inhibitor	log Kp (cm/s)
MMV003461	1	High	No	No	Yes	Yes	Yes	No	Yes	-4.52
MMV1804190	2	High	No	Yes	Yes	Yes	Yes	Yes	Yes	-5.45
MMV003140	3	High	Yes	Yes	Yes	No	Yes	Yes	Yes	-5.57
MMV1804185	4	Low	No	No	Yes	Yes	Yes	Yes	Yes	-6.28
MMV637528	5	High	No	Yes	Yes	Yes	Yes	Yes	Yes	-6.59
MMV662539	6	High	No	No	Yes	No	No	No	No	-7.37
MMV690777	7	High	No	Yes	No	Yes	No	Yes	No	-5.09
MMV001860	8	-	-	-	-	-	-	-	-	-
MMV010306	9	Low	No	No	Yes	Yes	Yes	Yes	Yes	-6.25
MMV1804194	10	High	No	Yes	No	No	Yes	Yes	Yes	-6.33
MMV1804175	11	High	Yes	No	No	Yes	Yes	Yes	Yes	-5.27
MMV1804174	12	High	No	Yes	No	Yes	Yes	Yes	Yes	-6.66
MMV003277	13	High	No	No	No	No	No	No	No	-5.37
MMV001681	14	High	Yes	Yes	No	No	Yes	Yes	No	-5.04
MMV000068	15	Low	No	Yes	No	No	No	No	No	-8.83
MMV638007	16	Low	No	Yes	No	Yes	No	Yes	Yes	-3.66
MMV637897	17	Low	No	Yes	No	No	No	No	No	-7.6
MMV007474	18	High	No	No	No	No	No	No	No	-5.51
MMV1804247	19	-	-	-	-	-	-	-	-	-
MMV1804250	20	High	No	No	No	No	No	No	No	-5.51
MMV001428	21	High	Yes	Yes	Yes	No	No	Yes	No	-4.9
MMV083882	22	High	No	No	Yes	Yes	Yes	Yes	No	-5.31
MMV1804354	23	High	Yes	Yes	No	No	No	Yes	No	-4.7
MMV1804359	24	High	No	Yes	No	Yes	Yes	Yes	Yes	-7.57
MMV000031	25	High	No	No	No	No	No	No	No	-7.63
MMV1804479	26	Low	No	Yes	No	No	Yes	Yes	No	-4.92
MMV892669	27	-	-	-	-	-	-	-	-	-
MMV1804412	28	High	Yes	Yes	No	No	No	Yes	Yes	-5.9
MMV002137	29	High	No	Yes	No	Yes	No	Yes	No	-4.64
Pyrimethamine[1]	30	High	Yes	No	Yes	Yes	No	No	Yes	-5.91

[1]Positive control; [2]Gastrointestinal; [3]Blood-brain barrier; [4]P-glycoprotein substrate.
