# Supplementary material for: Repurposing the Medicines for Malaria Venture’s COVID Box to discover potent inhibitors of Toxoplasma gondii, and in vivo efficacy evaluation of almitrine bismesylate (MMV1804175) in chronically infected mice
Source: PLoS One. 2023 Jul 7;18(7):e0288335. doi: 10.1371/journal.pone.0288335 (PMC10328330; doi:10.1371/journal.pone.0288335)
Supplement: S5 Table — (PDF) [file pone.0288335.s005.pdf]

MMV Code	ID	Druglikeness						Medicinal Chemistry			
		Lipinski violations	Ghose violations	Veber violations	Egan violations	Muegge violations	Bioavailability Score	PAINS alerts	Brenk alerts	Leadlikeness violations	Synthetic Accessibility
MMV003461	1	0	0	0	0	1	0.55	0	2	1	2.01
MMV1804190	2	2	3	0	0	1	0.17	0	0	2	4.86
MMV003140	3	0	0	0	0	0	0.55	0	0	3	3.1
MMV1804185	4	0	2	0	1	0	0.55	0	0	3	3.04
MMV637528	5	3	3	1	0	2	0.17	2	0	3	5.77
MMV662539	6	0	1	0	0	1	0.55	0	1	1	1.84
MMV690777	7	0	1	0	1	1	0.55	0	0	2	3.81
MMV001860	8	-	-	-	-	-	-	-	-	-	-
MMV010306	9	0	1	0	1	0	0.55	0	0	3	2.87
MMV1804194	10	1	3	1	0	2	0.55	0	3	3	5.38
MMV1804175	11	0	1	0	0	1	0.55	0	1	3	3.42
MMV1804174	12	1	2	0	0	0	0.55	0	0	2	3.87
MMV003277	13	1	4	0	0	2	0.55	0	0	2	7.01
MMV001681	14	1	1	0	0	1	0.55	0	0	2	3.88
MMV000068	15	1	1	1	1	2	0.11	0	1	1	5.17
MMV638007	16	1	1	0	1	1	0.55	0	2	3	3.18
MMV637897	17	2	4	1	1	4	0.17	0	2	2	10
MMV007474	18	1	3	0	0	2	0.55	0	0	2	6.89
MMV1804247	19	-	-	-	-	-	-	-	-	-	-
MMV1804250	20	1	3	0	0	2	0.55	0	0	2	6.89
MMV001428	21	1	0	0	0	1	0.55	0	0	2	3.9
MMV083882	22	0	0	0	0	0	0.56	0	1	1	2.43
MMV1804354	23	0	0	0	0	1	0.55	0	0	2	3.89
MMV1804359	24	0	0	1	0	0	0.55	0	0	2	4.14
MMV000031	25	0	0	0	0	0	0.55	0	1	0	3.17
MMV1804479	26	2	3	0	1	1	0.17	0	0	2	4.18
MMV892669	27	-	-	-	-	-	-	-	-	-	-
MMV1804412	28	0	0	0	0	0	0.55	0	0	2	4.24
MMV002137	29	1	2	0	1	1	0.55	0	0	2	3.2
Pyrimethamine[1]	30	0	0	0	0	0	0.55	0	0	1	2.43

[1]Positive control.
