# Supplementary figures and images for: Repurposing the Medicines for Malaria Venture’s COVID Box to discover potent inhibitors of Toxoplasma gondii, and in vivo efficacy evaluation of almitrine bismesylate (MMV1804175) in chronically infected mice
Source: PLoS One. 2023 Jul 7;18(7):e0288335. doi: 10.1371/journal.pone.0288335 (PMC10328330; doi:10.1371/journal.pone.0288335)

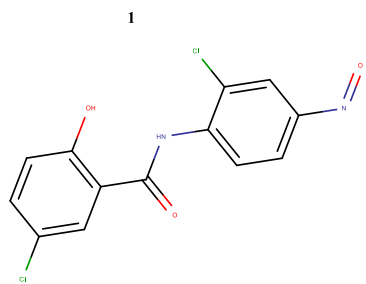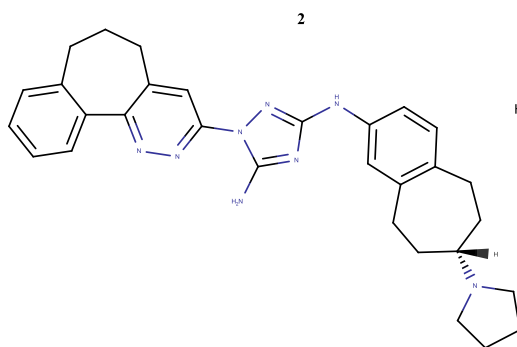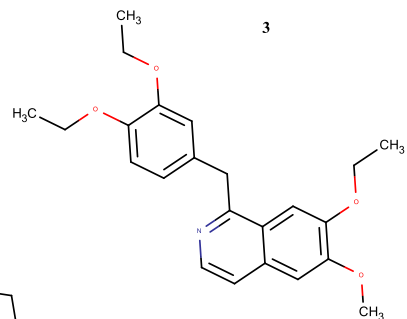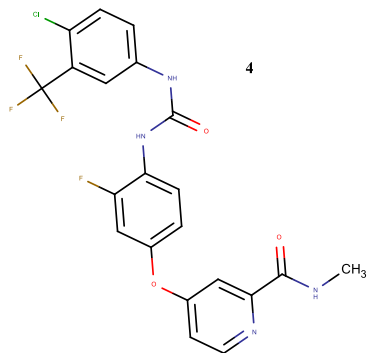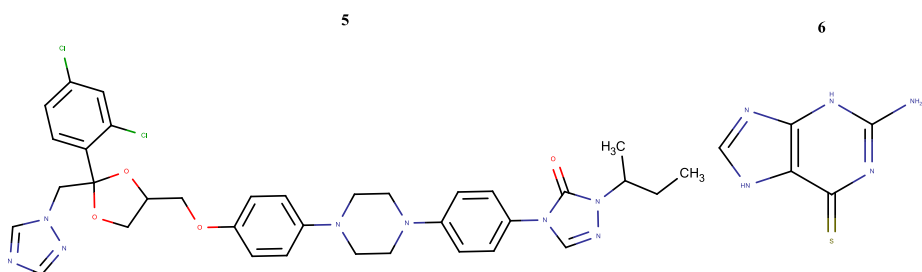

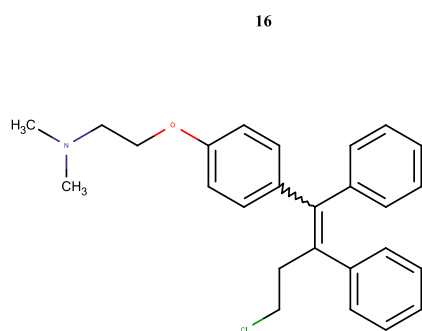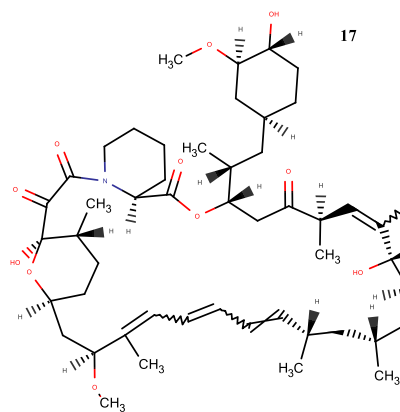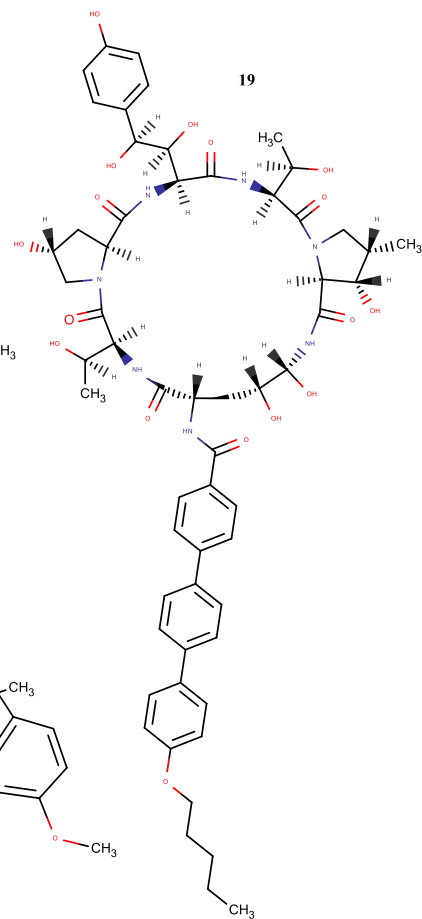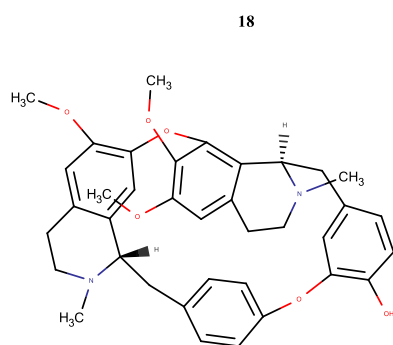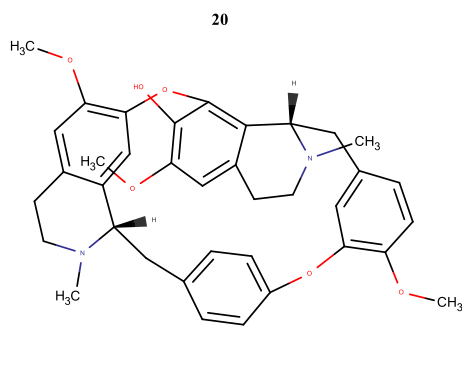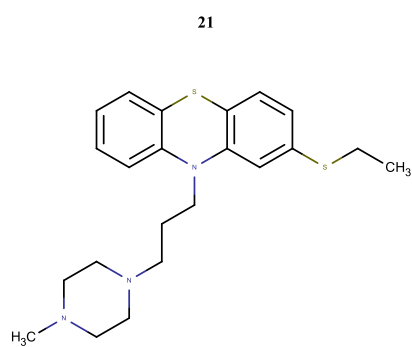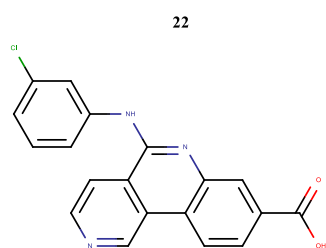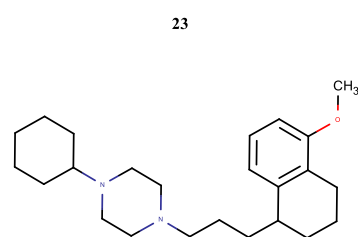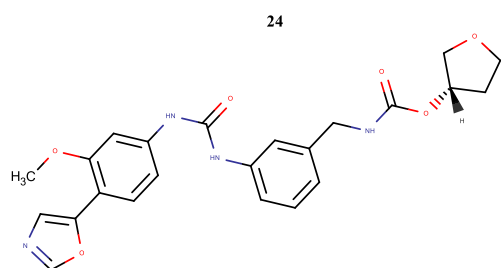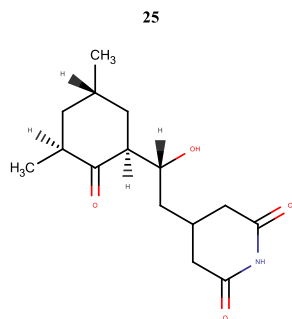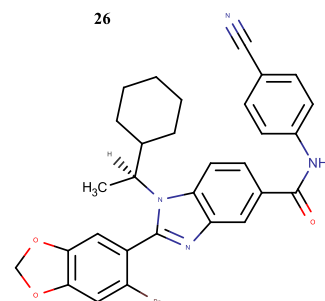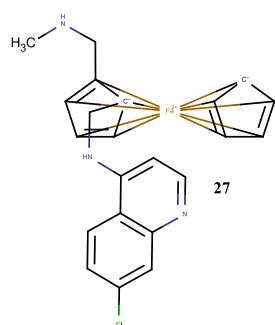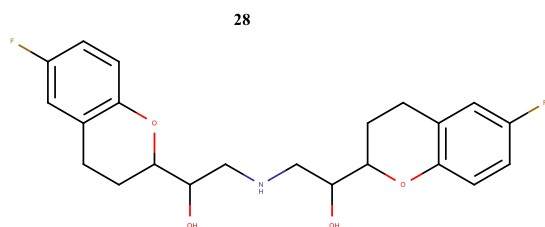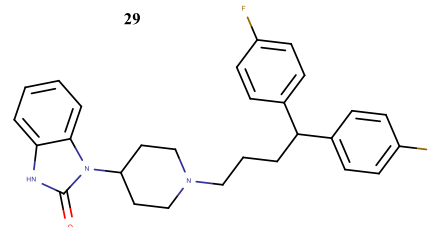

Supplement: S1 Fig — (PDF) [file pone.0288335.s006.pdf]
